# A Qualitative Study of Parental Perceptions of Baby Food Pouches: A Netnographic Analysis

**DOI:** 10.3390/nu14153248

**Published:** 2022-08-08

**Authors:** Madeleine Rowan, Miranda Mirosa, Anne-Louise M. Heath, Ioanna Katiforis, Rachael W. Taylor, Sheila A. Skeaff

**Affiliations:** 1 Department of Human Nutrition, University of Otago, Dunedin 9054, New Zealand; 2 Department of Food Science, University of Otago, Dunedin 9054, New Zealand; 3 Department of Medicine, University of Otago, Dunedin 9054, New Zealand

**Keywords:** complementary feeding, food pouches, infants, parenting forums, qualitative research, netnography, weaning

## Abstract

Globally, a recent phenomenon in complementary feeding is the use of squeezable baby food pouches. However, some health agencies have raised concerns about their possible long-term health effects. The aim of this study was to describe parental perceptions of the use of baby food pouches during complementary feeding (i.e., the transition from an entirely milk-based diet to solid foods) using a netnographic analysis of discussions on publicly available forums. In this study, the community was parents of young children. Six parenting forums were identified through a Google search using defined selection criteria. Discussion threads relating to baby food pouches were collected and imported into NVivo12 for thematic analysis via inductive reasoning. Perceptions of baby food pouches fell within two broad categories—benefits and concerns. The most commonly reported themes related to benefits were: convenience, health, baby enjoys, variety, and cost; whereas the most common concerns reported were: health, cost, lack of dietary exposure, dependence, and waste. Many parents reported both benefits and concerns. Once research has determined the long-term effect of using pouches on infants’ health regarding eating habits, nutritional status, growth, and development, the findings of this study can inform educational strategies to either encourage or discourage their use.

## 1. Introduction

Navigating complementary feeding, the transition from an entirely milk diet to solid food, can be daunting for parents. Many new challenges occur during this stage, leading parents to access advice on complementary feeding including what products to use, and how to provide their baby with food in a way that best suits the family’s lifestyle. A relatively recent phenomenon in complementary feeding is the increased use of squeezable baby food pouches. Globally, the unit sales of baby food pouches increased from 1.2 billion in 2015 to 2.2 billion in 2021 [1]. To date there is no research on the consumption of these food products. It has been proposed that parents or caregivers may choose to offer these squeeze pouches for convenience, ease of use, and to allow children to self-feed [2]. On the other hand, it has been suggested that feeding with squeeze pouches is an expensive alternative to making purées at home, and that food in pouches is less likely to provide the sensory experience of seeing, smelling, and touching new foods which may result in increased feeding difficulties [3,4]. To date, these questions have not been investigated in research studies, yet these opposing perspectives can make the use of pouches polarizing.

Today, parents can access online the type of information and support they would, in the past, have received from parents and friends [5]. A recent study reported that 94% of Australian parents with young children accessed the internet every day and 45% sourced parenting information from social networking sites (Facebook, Twitter, forums) [6]. Al-though virtual communities on social networking sites interact through the internet, they function very similarly to offline communities [7], however, in online spaces, parents can easily express their experiences and opinions with fewer inhibitions and greater honesty, due to the anonymity offered by the internet [8]. Given the accessibility and wealth of online parenting discussions now available, netnography, or online ethnography, is an innovative methodology which uses internet-based communications on social networking sites as a data source to understand a community [9] and to generate data about issues of relevance to parents, including food and nutrition [6]. Of note is the call by Ottrey et al. [10] to generate more ethnographic work within the field of nutrition. The aim of this study was to describe parental perceptions of the use of baby food pouches using thematic and content analysis of netnographic data from discussions on publicly available forums.

## 2. Materials and Methods

This was a netnographic study using internet-based communications on social networking sites as a data source to understand a community; in this study, the community was parents of young children. Discussion forums relevant to parents undertaking complementary feeding were found through a Google search. This search was refined to six websites from which 78 threads relating to squeezable baby food pouches were captured for thematic analysis using NVivo12.

To locate parenting discussion forums, the phrases “parenting forum”, “mum forum”, “motherhood discussion board”, and “motherhood forum” were typed into the commercial search engine Google. Advertisements were ignored, and the first 20 websites generated after each search were collected, resulting in a sample of 80 potential websites.

From the 80 potential websites, forums were included if they met the following criteria recommended by Kozinets [11]: (i) publicly available with no membership or password protection; (ii) the name of the website or description of the forum suggested it provided relevant discussions; (iii) discussions have been created within a timeframe relevant to the topic [12] written in English. To meet criterion (ii) discussions were identified for parents with children aged 6–24 months by forum titles such as “Babies: 0–12 months” or “introducing solids”; to meet criterion (iii) discussions between 2014 and 2019 were captured to align with the emergence of pouches. This refined the potential websites down to the six which were used for analysis. All forums contained a search engine where discussions threads were manually searched for using the key word “pouch”.

This study focused on archival data, which have been defined as comprising anything the researcher can gather from the web that is not a part of his or her involvement to create or prompt the creation of data, this constitutes a “cultural baseline” of the online community as it describes what is already occurring prior to researcher observation [11]. All data were collected between 26 November and 2 December 2019.

As there were insufficient data on the demographics of forum participants across the six websites, this information has not been reported. Other netnographic studies [8] have also not included demographic data due to anonymous online user profiles.

There is some debate in netnographic research around the ethical issue of informed consent [9,13,14]. The current study followed the protocol of Lynch [14] to only include public discussions using the following inclusion criteria: message boards were not required to have a membership, registration, sign-in, or a password; and the boards were publicly accessible through a popular internet search engine. This ensures that participants are aware that what they post is publicly available.

While the data collected in this study did contain personal opinions, these were not about controversial or sensitive topics that could result in harm for the participants if quoted. Despite this, the names of the forum websites and the members who participated in the forums captured for this study are not reported, ensuring no identifying information is provided. Members who participated in threads are quoted in this paper without any label even where pseudonyms were used. This is to protect all the participants, as Bruckman [15] suggests that pseudonyms function similarly to real names and should be treated with equal caution.

The entire webpage of the threads were captured into the software NVivo12 [16]. Consistent with the thematic analysis approach outlined by Braun and Clarke [17], the first phase of analysis began with data familiarisation by reading and re-reading all data items and making reflective notes. Following familiarisation, coding began [17,18]. Initially themes were created via inductive reasoning, at the point when there was only accumulation of existing themes and no new themes, theoretical saturation was reached [19]. Through cycles of refinement and grouping, a final coding scheme was created. Counts of themes in NVivo12 were used for content analysis. A first level theme is a main over-arching theme, second level themes are subthemes within first level themes, and third level themes are subthemes within second level themes [17].

The components of a forum include the original comment or query made under the title, followed by a series of comments in reply. The original comment and all those which follow make up the total thread, which gives an overall discussion. The total thread was not coded in its entirety because it usually contained a range of views. Instead, statements within individual comments were coded. Purely behavioural or emotive statements were not coded. For example, the first part of the quote “*I’ve never bought a pouch and I don’t really like them*” does not give any thematic insight. Rather, themes were identified based on the reason the parent provided for considering that baby food pouches provided benefit or were a concern. For example, the concern expressed in the following quote was coded into the theme “cost”: *“I’ve never bought a pouch and I don’t really like them. A big reason is the cost! I just can’t justify it with all the other easy and cheaper options”*.

All the data were collected and coded by the lead researcher (M.R). A second researcher (I.K.) independently coded 80% of the dataset, using the final coding scheme. A Cohens kappa value was calculated to establish inter-rater agreement. Across the discussion threads, there was almost perfect agreement between the raters (k = 0.92) [20].

## 3. Results

Following the inclusion criteria recommended by Kozinets [9], 6 of the initial 80 forum websites were included in the final sample. There were 35 websites which did not include a forum aspect (news articles, journal articles, parenting websites) and 14 duplicates reducing the prospective sample to 31. Three websites were not written in English, 7 websites were password protected, 5 websites had not been active past the year 2014, and 12 websites did not relate to children at the stage of life of interest (6 to 24 months). Within the final 6 websites, 78 discussion threads were identified that were relevant to baby food pouches.

Table 1 summarizes the attitudes coded as “benefit” themes (only themes which were expressed at least 10 times are reported). A total of 22 benefit themes were identified through data analysis, 8 first level themes and 14 second or third level themes. Convenience was the theme related to benefits that appeared most often in the data, suggesting it may be a key determinant influencing use. It encompassed many time and effort considerations which are of importance to parents with young children. Health effects, such as the nutritional value, use while navigating allergies, and how much the baby enjoys having food in pouches were also reported by many parents to be benefits. Table 1 also summarizes the attitudes coded as “concern” themes (again, only themes which were expressed at least 10 times are reported). Seventeen concern themes were identified through thematic analysis with 9 being first level themes and 8 being second or third level themes. Benefits (i.e., 628 counts) were reported almost twice as often as concerns (i.e., 337 counts). Themes with <10 counts coded as benefits at first level included “less food waste” (8 counts) and “takes up less space” (7 counts), at second level was “great for allergies” (5 counts), and a third level was “more healthy than own foods” (4 counts). A concern about “allergic reaction” was a third level theme with 5 counts.

The key finding of the thematic analysis was that there were a wide variety of perceptions of baby food pouches within the categories of benefits and concerns (Table 2). It was clear from the analysis that parents were invested in the complementary feeding transition, and it seemed there were a range of influencing factors which resulted in more nuanced, rather than clearly divided, opinions. Many participants noted that they could see both benefits and concerns from the use of the pouches as this participant quote illustrates (coded as “Easier to feed fruit and vegetables” and “Dependence”): *[what are your thoughts on pouches these days?] “Well, it’s the only way my kid would eat any fruits or vegetables for a long time. The pediatrician said, like all things, as long as they’re given in moderation. He’s finally opening up to real fruits and vegetables, so we are limiting the pouches more and more, but I don’t think they’re necessarily “bad” unless you’re giving them in place of more nutritious food.”*

Many parents were open to using the pouches in certain situations, especially when away from home, if they were short on time, or to encourage fruit and vegetable intake, but not many parents recommended using them as the sole source of food for an infant. Interestingly, the substantially higher counts for benefits compared to concerns suggests that the study population were, overall, in favour of the pouches.

## 4. Discussion

The goal of this netnographic study was to determine parental perceptions of the use of baby food pouches during complementary feeding. The key finding was that parents expressed a wide range of opinions regarding baby food pouches that fell under the broad categories of benefits and concerns. Interestingly, health and cost appeared as both a benefit and a concern to parents, whereas themes specific to benefits were convenience, baby enjoys, and variety, and those only appearing as a concern included lack of dietary exposure, dependence, and waste.

The finding that health was the predominant concern is in line with the limited previous research. The survey by Seaman et al. [21] on choice of weaning foods found that 30% of mothers with 18-month-old babies believed commercial infant food had low nutritional value with nearly half of these mothers stating that commercial infant foods had a high sugar content. Beauregard et al. [22] found squeeze pouches from 3 of the 5 food groups commercially available in the USA in 2015 were more likely to contain added sugars compared to other packaging types. In another USA study, Moding et al. [23] found that baby food pouches contained significantly more total sugar per serving compared to baby foods in jars, packs, and other containers (although not per 100 g). Interestingly, a recent study of infant baby pouches in New Zealand reported significantly higher median total sugar content for pouches than their non-pouch equivalents (per 100 g), but that, on average, squeeze pouches did not contain any free or added sugar [24]. As discussed in that paper, these findings depend on the definition of free or added sugars, as well as on the food supply. Regularly feeding an infant with high sugar pouches may increase the risk of overfeeding because of their palatability and result in excessive infant weight gain, which has been associated with an increased risk [12] of obesity later in life. The Nutrition Commission of the German Society for Pediatrics and Adolescent Medicine also expressed a concern about the increased risk of dental caries and their position paper discouraged the use of baby food pouches [25]. However, no research to date has reported on the impact of using baby food pouches on infant growth or dental health.

In the current study, thematic analysis suggested that a greater proportion of parental comments within these online communities were related to the benefits of using baby food pouches, than concerns. In particular, the most frequently expressed benefit for baby food pouches was convenience. Although studies have reported a negative reaction by some parents towards pre-prepared infant foods [26], increased use of commercial infant food is a consequence of limited time and resource constraints experienced by busy parents who often work and may have poor cooking skills [27]. About a decade ago, manufacturers of infant foods began to shift away from glass jars to plastic packaging and pouches [28]. For the manufacturer, plastic was easier and cheaper to transport because it weighs less than glass; furthermore, it does not break. For parents, pouches can also be heated in a microwave (although this is not recommended because of the potential for hot spots to develop than can cause burns), allow children to “self-feed”, and can be resealed if not completed in a single sitting (although it is recommended that food not eaten at the end of the meal that has come into contact with the infant’s mouth is thrown out) [29]. Given this, it is no wonder that the sales of these products are rising [2], and in many countries, pouches are the predominant form of packaged infant foods. Research is beginning to emerge that suggests parents may need to be more cautious about their use [22]; however, these findings are not necessarily unanimous [24], and there is not yet the necessary scientific evidence to discourage, or indeed promote, the use of baby food pouches. Studies should be undertaken to understand the reasons for the popularity of baby food pouches, with the current study demonstrating a range of views that would justify the expense of a large face-to-face study with families. Further research is also needed to ascertain the long-term effect of the use of pouches on the health of infants regarding eating habits, nutritional status, growth and development [30]. Given the range of attitudes of parents towards baby food pouches identified in this study, further qualitative research, using either individual interviews or focus groups, would be valuable to explore how parents negotiate their own attitudes towards baby food pouches with those of other parents, and health professionals.

Of interest to these authors was the relative lack of parental discussion about packaging concerns related to pouches. As the discussion of single use plastics is growing in other areas [31], it may be advisable that manufacturers use biodegradable plastics to avoid potential future negative feedback given concerns about sustainable food choices and climate change. It is surprising to note that some parents (5 counts) associated baby food pouches with less waste (i.e., a benefit) although there were considerably more parents (27 counts) who expressed a concern about waste. Interestingly, the waste-related benefits of pouches tended to be around food waste, while concerns were more around packaging; baby food pouches typically utilise multi-material multilayer packaging which is difficult to recycle [32].

Previous studies have noted other influences on baby food choice to include advertising and brand trustworthiness [21,30]. Many of the discussion threads in the present study did include brand names and talk around which products were the best to use but this was not included in the coding scheme of the present study because it did not provide much thematic insight as to the overall perception of the product. However, this could be an opportunity for future research to see if brand recognition and trustworthiness is an influencing factor for pouch use. The current research highlights three key strengths of netnography as a methodology. First, the method is unobtrusive and objective. Second, it allows researchers to exploit a freely accessible wealth of data to rapidly gather an understanding of a community’s baseline views [11]. Finally, at the time this research was undertaken, only a single netnography study in the field of nutrition and dietetics [14] had been published; the findings of both that study and the current study confirm that it is a method well suited to understanding parents views on feeding their children

The limitations of this study should also be noted. The extent to which this sample of online posts is representative of all parents is unknown. Although the socio-demographic characteristics and geographical location of the parents are not available, this information is less relevant for this type of analysis because social media is not bound by these limits. We purposefully chose websites any parent could access via the internet, both to post comments and to read other people’s comments. Parents could have commented on multiple websites; however, it was assumed they would express the same attitudes which would fall under the same theme or subtheme. In the current sample, only limited posts shared demographic information such as gender, age, and number of the children, while socioeconomic status and cultural background were never indicated. Additionally, parents who do not have strong opinions regarding pouches may not engage in these online forums.

## 5. Conclusions

The wide variety of ways in which parents in this study discussed the use of baby food pouches illustrates the complex and sometimes polarising perceptions towards this food product. There were several themes which fitted under the broad categories of benefits and concerns, with convenience seeming to be a primary determinant of use despite concerns about health. Further research is urgently needed in the area, particularly in light of rising sales of these products. Once research has determined the long-term effect of using pouches on infant’s health regarding eating habits, nutritional status, growth and development, the findings of this study can inform educational strategies to either encourage or discourage their use.

## Figures and Tables

**Table 1:** Parental attitudes regarding benefits and concerns of baby food pouch use across forum thread.

Benefits		Concerns	
Theme ^1^	Count ^2^	Theme ^1^	Count ^2^
**Convenience**	**349**	**Health**	**121**
Away from Home	122	Low Nutritional Value	71
Time	42	*High sugar*	65
Good snack	36	*Processed*	20
Self-feeding	28	Delays oral motor development	25
Stays fresh	17	Preservatives	19
Less mess	11	**Cost**	69
**Health**	**146**	**Lack of dietary exposure**	**39**
Good nutrition	63	Flavours and tastes	24
*Good ingredients*	32	Textures	23
*High in nutrients*	19	**Dependence**	**36**
Easier to feed fruit and vegs	36	**Waste**	**27**
Organic	21	**Hygiene/safety**	**23**
Sickness	10	**Unappealing taste**	**12**
**Baby enjoys**	**55**	**Mess**	**10**
**Variety**	**36**	Total	337
**Cost**	**16**		
**Well controlled production**	**11**		
Total	628		

^1^ Themes are presented as follows; **level one theme**, under this are the level two themes, followed by *level three themes.* Not all level one themes had level two and level three themes. Only themes with ≥10 counts are included in this table. ^2^ Count is the number of times the theme was referenced across 78 forum threads.

**Table 2:** Categories, themes, and illustrative quotes related to parental perceptions of the use of squeezable baby food pouches via parenting discussion threads as identified by netnographic analysis ^1^.

Benefits		
Theme	Definition	Illustrative Quote(s)
**Convenience**	States or implies convenience with no other explanation	*“…to be honest I like that they are ready to go”* *“I use them occasionally for convenience.”*
Away from Home	Could be used when away from home	*“I did use a few store bought pantry pouches as they were extremely convenient for camping or trips out and around the farm”*
Time	Takes less time	*“But I am back at work 5 days and honestly don’t know how mums find the time to give everything homemade”*
Good snack	Use as a snack in-between meals or to “supplement” diet	*“Is anyone else using store bought baby food for snacks and to supplement bubs diet?” * *“…fruity ones usually go as two snack portions”*
Self-feeding	Baby can feed themself	*“I tried to make purée for her she would rarely eat it. Hated being fed from a spoon, it made life a little bit difficult. One day I picked up some pouches and down she sucked it!”*
Stays fresh	No need to be kept cool and/or be heated up. Have a long shelf life	*“But I use pouches etc out and about, they are...safe to keep at* *room temp if unopened”* *“Pouches are packed in a way that they can sit for ages on the shelf”*
Less mess	Less mess	*“no fuss and mess”*
**Health**	States or implies healthy with no other explanation	*“They’re usually pretty healthy”*
Good nutrition	States or implies nutritional value	*“There is nothing wrong with them nutritionally”*
Good ingredients	Good ingredients	*“The pouches can be kept at room temp and I’ve yet to be unsatisfied with the ingredients list on a product, even the yoghurt ones!”*
High in nutrients	High in nutrients	*“I can pretty much count on him downing two pouches and I can sleep easy knowing hes got a belly full of healthy vitamins*
More healthy than own foods	More healthy than the food being eaten by the rest of the family	*“or if we are out or getting takeaway—I can have peace of mind knowing she is having veges etc rather than deep fried chips*
Easier to feed fruit and vegs	Easier to get baby to eat fruits and vegetables	*“Mine flat out will not eat fruit unless its in a smooth or a pouch. Buy all the pouches”*
Organic	Organic	*“If a pouch claims that it is pure, organic broccoli with nothing else added, then I can’t see how this is so massively different from me buying, steaming and pureeing the same.”*
Sickness	Gets baby to eat when feeling unless	*“14 month old is teething. He went from being a pretty impressive eater to only consume blueberries and pouches. * *Pouch it up, little dude”*
Great for allergies	Easy to identify ingredients Food parents won’t cook due to allergies	*“Another use for jars/pouches for me is that I use them to feed DD pulses which is a food group I’m allergic to and don’t want to be cooking myself.”*
**Baby enjoys**	Mentions baby enjoying/preferring pouches	*“i feed mine only the food pouches or jars it works for us:) * *i did did try to get her to eat home-made but she just didn’t * *like my cooking lol”*
**Variety**	Provides baby with wider range of flavours than family foods/purées	*“offer a wide range of foods that we would never normal eat”* *“foods from all different cuisines and so many interesting flavours”*
**Cost**	Cost less	*“have made some purées myself but in all honesty to do the variety of pouches would take me an age and to me more costly…”*
**Well controlled ** **production**	Can trust the product due to high standards	*“All baby food for sale has to meet the same food safety standards designed to assure customers of safety and quality. * *The levels of pesticides…all strictly regulated.”*
**Less waste**	Both packaging and food waste	*“He also ate such tiny amounts the pouches were great as he could have as much or little as he wanted with minimal waste.”*
**Takes up less space**	Takes up less space	*“They are so easy to pack” “Plus our freezer just doesn’t have enough room...we too are switching to jars/pouches…”*
**Concerns**		
**Health**	States or implies not healthy with no other explanation	*“The pouch labels are a marketing ploy. The food isn’t any * *healthier because of it. Homemade food is always better for baby.”*
Low Nutritional Value	Concerns about nutritional quality but no mention of sugars or processing	*“Only ingredients are listed in order of which there is the most of and the first ingredient is often rice or maize. if you were making it yourself it would probably… have no filler”*
High sugar	High sugar	*“Another thing is the mixes can have a lot of sugar as the ratio fruit to beg is high” “often vegetable ones have apple or pear added to sweeten them up.”*
Processed	Heat processing diminshes nutrients, is still processed food like those for adults	*“I read … the fruit and veggie ones are heated up to around * *250 degrees when they are sealed into the pouches which pretty much destroys any nutritional goodness”*
Delays oral motor development	Delays oral motor development	*“will never allow him to suck on them as it can be bad for * *their development and teeth.” “It’s really important for them to experience textures and tastes at that age....”*
Preservatives	Concerns about preservatives	*“I guess because anything that’s in a pouch on a supermarket shelf has additives and preservatives in it to give it shelf life where as home prepared food won’t have any of that”*
Allergic reaction	Concerns about allergic reactions	*“we were told to try one food at a time for allergy reasons, and a lot of store bought baby food is a mix of two or more”*
**Cost**	Higher cost	*“I’ve never bought a pouch. A big reason is the cost! I just can’t justify it with all the other easy and cheaper options”*
**Lack of dietary exposure**	No explicit mention of flavour/texture but negative comments about dietary exposure	*“…[homemade purees instead of pouches] exposes them more to what their diet will be”*
Flavours and tastes	Bland, doesn’t give a range of flavours of home food	*“I don’t think they encourage good long term eating habits, * *the savoury meals are often quite bland…and stewed and pureed fruits I’ve made are never as sweet as pouches”*
Textures	Mention of texture or sensory	*“They don’t give baby’s/toddlers the full sensory information about food. (An apple pureed in a pouch doesn’t teach what an apple looks & feels like).”*
**Dependence**	Is the only thing that baby will eat, creates fussy eaters	*“My bub used to be great with food, eat everything and anything given to her. Now she will only eat baby food pouches. ”*
**Waste**	Packaging, food waste	*“I just can’t get behind the amount of waste we’re dumping* *after one use” “also think it is harder to get everything out of the pouch so there is more waste.”*
**Hygeine/safety**	Mould scares, distrust of not being able to contents	*“There have been instances of mold in them… and since you can’t see it, baby gets it”* *“I don’t trust them unless I put them in a bowl and look at them”*
**Unappealing taste**	Parent perceives the taste to be unappealing	*“but also cos my kids won’t have a bar of them and when I’ve tasted them I can kinda see why”*
**Mess**	Mess	*“Much before 18 months they were way too messy for him * *to self feed with”*

^1^ Themes are presented as follows; **level one theme**, under this are the level two themes, followed by *level three themes.* Not all level one themes had level two and level three themes. Only themes with ≥10 counts are included in this table.

## Data Availability

Data presented in this study can be obtained, upon reasonable request, from the corresponding author.
